# Supplementary material for: Fungal sensing by dectin-1 directs the non-pathogenic polarization of TH17 cells through balanced type I IFN responses in human DCs
Source: Nat Immunol. 2022 Dec 1;23(12):1735–48. doi: 10.1038/s41590-022-01348-2 (PMC9747615; doi:10.1038/s41590-022-01348-2)
Supplement: Supplementary file 1 — Supplementary Information [file 41590_2022_1348_MOESM1_ESM.pdf]

nature immunology

Article https://doi.org/10.1038/s41590-022-01348-2

Fungal sensing by dectin-1 directs the non-pathogenic polarization of T_H17 cells through balanced type I IFN responses in human DCs

In the format provided by the authors and unedited

Expression primer sequences

Gene	Forward primer	Reverse primer
IFNB	ACAGACTTACAGGTTACCTCCGAAAC	CATCTGCTGGTTGAAGAATGCTT
MX1	TTCAGCACCTGATGGCCTATC	GTACGTCTGGAGCATGAAGAACTG
ISG15	TTTGCCAGTACAGGAGCTTGTG	GGGTGATCTGCGCCTTCA
TRIM22	CTGTGCCTCCCTGTCGTATTG	GAGTGCTCCGTGGTTTGTGAC
IRF7	GCTCCCCACGCTATACCATCTAC	GCCAGGGTTCCAGCTTCAC
IFNG	CTTGAATGTCCAACGCAAAGC	TGGCGACAGTTCAGCCATC
TBX21	ACCCAGATGATTGTGCTCCAG	CCTCAACGATATGCAGCCG
IL1R1	AGCCCAGCTAATGAGACAATGG	TGGCCGGTGACATTACAGATC
CXCL3	AGTGTGAATGTAAGGTCCCCCG	GCTTTCTTCCCATTCTTGAGTGTG
CCL3	CTGCCCTTGCTGTCCTCCT	GCAAGTGATGCAGAGAACTGGTT
CSF2	CCTGTGCAACCCAGATTATCAC	CAAGCAGAAAGTCCTTCAGGTTC
IL22	CACTGCAGGCTTGACAAGTCC	AGCATGAAGGTGCGGTTGG
CCL5	GCTGTCATCCTCATTGCTACTGC	TGTGGTGTCCGAGGAATATGG
IL17A	TCCAGCAAGAGATCCTGGTCC	CCAGCCGGAAGGAGTTGG
IL17F	AAGGCTGCTCTGTTTCTTTCCAG	TGCACATGGTGGATGACAGG
RORC	GTGGTGCTGGTTAGGATGTGC	CCTTCAAAAAAGACCGTGCG
IL10	GAGGCTACGGCGCTGTCAT	CCACGGCCTTGCTCTTGTT
MAF	CTGCAGCAAGTCGACCACC	TGTACGCGTCCCTCTCGC
PTGDS	TGTCCATGTGCAAGTCTGTGG	CACTGGTTTTTCCTGAGGAAGG
SYK	CCAGAGACAACAACGGCTCC	TGTCGATGCGATAGTGCAGC
CARD9	CATGTCGGACTACGAGAACGAT	CAGGTAAGGTGTGATGCGTGA
BCL10	ATGGAGCCACGAACAACCTCT	TCGTGCTGGATTCTCCTTCTG
MALT1	GACCCATTCCATGGTGTTTACC	AATAAATGCATCTGGAGTCCGG
LSP1	TACAGGCTCAGTCTGCGGC	GGCTTTTCTTGCTCATGTCTCC
RAF1	GGTGATAGTGGAGTCCCAGCA	TCAGATGAGGGACTGGAGGTG
IRF1	TTATACAGTGCCTTGCTCGGC	AGGCGCTCACACTTCCCTC
IRF5	AGCCAGGACGGAGATAACACC	GGATCGGCTTCATCCACG
ITGAV	GGTTATTCTGTGGCTGTCGGAG	AAAGTCCTTGCTGCTCTTGGAAC
ITGB8	GCTTACCTGCACCGCTTGC	TCGCTAGGACGTTTTCCGC
BST2	TCACCATCAAGGCCAACAG	TGACATTGCGACACTCCATC
GAPDH	CCATGTTCGTCATGGGTGTG	GGTGCTAAGCAGTTGGTGGTG
ACTB	CGACAGGATGCAGAAGGAGA	CATCTGCTGGAAGGTGGACA

ChIP primer sequences

Gene (element)	Forward primer	Reverse primer
IFNB (ISRE)	TGACATAGGAAAACTGAAAGGGAG	GTCCTTTCTCCATGGGTATGG

Supplementary Table 1 | Primer sequences used for mRNA expression and ChIP analyses by real-time quantitative PCR.
