# Supplementary material for: Fungal sensing by dectin-1 directs the non-pathogenic polarization of TH17 cells through balanced type I IFN responses in human DCs
Source: Nat Immunol. 2022 Dec 1;23(12):1735–48. doi: 10.1038/s41590-022-01348-2 (PMC9747615; doi:10.1038/s41590-022-01348-2)
Supplement: Supplementary file 2 — Reporting Summary [file 41590_2022_1348_MOESM2_ESM.pdf]

nature research

Corresponding author(s): S.I. Gringhuis, T.B.H. Geijtenbeek

Last updated by author(s): Sep 25, 2022

Reporting Summary

Nature Research wishes to improve the reproducibility of the work that we publish. This form provides structure for consistency and transparency in reporting. For further information on Nature Research policies, see our Editorial Policies and the Editorial Policy Checklist.

Statistics

For all statistical analyses, confirm that the following items are present in the figure legend, table legend, main text, or Methods section.

n/a	Confirmed	
☐	☒	The exact sample size (*n*) for each experimental group/condition, given as a discrete number and unit of measurement
☐	☒	A statement on whether measurements were taken from distinct samples or whether the same sample was measured repeatedly
☐	☒	The statistical test(s) used AND whether they are one- or two-sided<br>*Only common tests should be described solely by name; describe more complex techniques in the Methods section.*
☒	☐	A description of all covariates tested
☒	☐	A description of any assumptions or corrections, such as tests of normality and adjustment for multiple comparisons
☐	☒	A full description of the statistical parameters including central tendency (e.g. means) or other basic estimates (e.g. regression coefficient) AND variation (e.g. standard deviation) or associated estimates of uncertainty (e.g. confidence intervals)
☐	☒	For null hypothesis testing, the test statistic (e.g. F, t, r) with confidence intervals, effect sizes, degrees of freedom and P value noted<br>*Give P values as exact values whenever suitable.*
☒	☐	For Bayesian analysis, information on the choice of priors and Markov chain Monte Carlo settings
☒	☐	For hierarchical and complex designs, identification of the appropriate level for tests and full reporting of outcomes
☒	☐	Estimates of effect sizes (e.g. Cohen's d, Pearson's r), indicating how they were calculated

Our web collection on statistics for biologists contains articles on many of the points above.

Software and code

Policy information about availability of computer code

Data collection	Microsoft Office Professional Plus 2019 - Excel
Data analysis	Microsoft Office Professional Plus 2019 - Excel, GraphPad Prism 8.3.1, FlowJo 10.8.1, Primer Express 2.0

For manuscripts utilizing custom algorithms or software that are central to the research but not yet described in published literature, software must be made available to editors and reviewers. We strongly encourage code deposition in a community repository (e.g. GitHub). See the Nature Research guidelines for submitting code & software for further information.

Data

Policy information about availability of data

All manuscripts must include a data availability statement. This statement should provide the following information, where applicable:

- Accession codes, unique identifiers, or web links for publicly available datasets
- A list of figures that have associated raw data
- A description of any restrictions on data availability

The authors declare that the data supporting the findings of this study are available within this paper and its source data files.

Field-specific reporting

Please select the one below that is the best fit for your research. If you are not sure, read the appropriate sections before making your selection.

☒ Life sciences ☐ Behavioural & social sciences ☐ Ecological, evolutionary & environmental sciences

For a reference copy of the document with all sections, see nature.com/documents/nr-reporting-summary-flat.pdf

Life sciences study design

All studies must disclose on these points even when the disclosure is negative.

Sample size	No statistical methods were used to predetermine sample size.
Data exclusions	No data were excluded. We did routinely screen our buffy coat donors before experiments for dectin-1 single nucleotide polymorphism rs16910526 using TaqMan SNP Genotyping Assays (Assay ID C_33748481_10; Applied Biosystems) and only used dectin-1 wild-type donors for experiments, unless otherwise indicated.
Replication	Typically, we performed all experiments at least 3 or more times to confirm robustness of biological observations, with a few exceptions when results were 'black and white' (no change or complete block).
Randomization	No randomization was used.
Blinding	Experiments were not blinded but kept as unbiased as possible.

Reporting for specific materials, systems and methods

We require information from authors about some types of materials, experimental systems and methods used in many studies. Here, indicate whether each material, system or method listed is relevant to your study. If you are not sure if a list item applies to your research, read the appropriate section before selecting a response.

Materials & experimental systems

n/a	Involved in the study	
☐	☒	Antibodies
☐	☒	Eukaryotic cell lines
☒	☐	Palaeontology and archaeology
☒	☐	Animals and other organisms
☐	☒	Human research participants
☒	☐	Clinical data
☒	☐	Dual use research of concern

Methods

n/a	Involved in the study	
☒	☐	ChIP-seq
☐	☒	Flow cytometry
☒	☐	MRI-based neuroimaging

Antibodies

Antibodies used

CD4 T cell isolation:
Alexa Fluor 488-conjugated anti-CD4 (1:50; clone RPA-T4, 300519, Biolegend)
APC-conjugated anti-CD45RA (1:50; clone HI100, 550855, BD Bioscience).
PE-conjugated anti-CD45RO-PE (200 µg/ml; clone UCHL1, R084301-2, Agilent)

During culture:
blocking antibodies against dectin-1 (20 µg/ml; clone #259931, MAB1859, R&D Systems)
blocking antibodies against IFNα/βR2 (20 µg/ml; clone MMHAR-2, PBL Assay Science)
anti-αvβ1 (10 µg/ml; clone #P5D2, MAB17781, R&D Systems)
anti-αvβ3 (10 µg/ml; clone #23C6, MAB3050, R&D Systems)
anti-αvβ5 (10 µg/ml; clone #P5H9, MAB2528, R&D Systems)
anti-αvβ6 (10 µg/ml; clone 10D5, ab77906, Abcam)
anti-αvβ8 (10 µg/ml; kind gift from S. L. Nishimura) (not commercially available)
mouse IgG1 isotype control (20 µg/ml; clone MOPC-21, 555746, BD Pharmingen)
mouse IgG2a isotype control (20 µg/ml; clone G155-178, 555571, BD Bioscience)
mouse IgG2b isotype control (20 µg/ml; clone #20116, MAB004, R&D Systems)
neutralizing antibodies against IL-1β (5 µg/ml, AF-201-NA, R&D Systems)
neutralizing antibodies against IL-23 (5 µg/ml, AF1716, R&D Systems)
neutralizing antibodies against IL-12 (5 µg/ml, AF-219-NA, R&D Systems)
neutralizing antibodies against IL-27 (5 µg/ml, AF2526, R&D Systems)
neutralizing antibodies TGFβRII (5 µg/ml, AF-241-NA, R&D Systems)
normal goat IgG (AB-108-C; R&D Systems) as a control

FACS staining:
anti-IRF1 (1:50; ab26109, Abcam)
anti-IRF5 (1:50; ab124792, Abcam)
anti-αv (1:50; AF1219, R&D Systems)
anti-β8 (1:50; ab80673, Abcam)
anti-BST2 (1:100; NIH AIDS Reagent program #11721)
PE-conjugated anti-rabbit (1:200; 711-116-152, Jackson Immunoresearch)
Alexa Fluor 488-conjugated anti-goat (1:400; A11055, Invitrogen)
FITC-conjugated anti-IFNβ (1:20; clone MMHB-3, 21400-3, PBL Assay Science)
FITC-conjugated IgG1κ isotype control mouse Ab (1:250; clone P3.6.2.8.1, 11-4714-81, eBioscience)
APC-conjugated anti-IL-17 (1:25; clone eBio64DEC17, 17-7179-42, eBioscience)
eFluor506-conjugated anti-IL-17 (1:50; clone eBio64DEC17, 69-7179-42, eBioscience)
FITC-conjugated anti-IFN-γ (1:5; clone 25723.11, 25723.11, BD)
PE-conjugated anti-IL-10 (1:10; clone JES3-9D7, 12-7108-82, Invitrogen)
Alexa Fluor 647-conjugated anti-RORγt (1:10; clone Q21-559, 563620, BD)
APC-conjugated anti-T-bet (1:10; clone 4B10, 644814, Biolegend)
eFluor660-conjugated anti-c-Maf (1:10; clone sym0F1, 50-9855-82, Invitrogen)
APC-conjugated anti-IL-1R1 (1:10; FAB269A, R&D Systems)
APC-conjugated anti-GM-CSF (1:10; clone BVD2-21C11, 502310, Biolegend)
Alexa Fluor 647-conjugated mouse IgG2b (1:5; clone 27-35, 558713, BD)
APC-conjugated mouse IgG1 (10 μg/ml; clone P3.6.2.8.1, 17-4714-42, Invitrogen)
eFluor660-conjugated mouse IgG2b (10 μg/ml; clone eBMG2b, 50-4732-82, Invitrogen),
APC-conjugated goat IgG (1:5; IC108A, R&D Systems)
APC-conjugated rat IgG2a (2.5 μg/ml; 402305, Biolegend)

ELISA:
anti-pan TGFβ (2 μg/ml, coating Ab; clone 1D11, MAB1835, R&D Systems)
biotinylated anti-TGFβ1 (0.2 μg/ml, detecting Ab; BAF240, R&D Systems)

Immunoblotting:
anti-IRF1 (1:1000; 8478, Cell Signaling)
anti-IRF3 (1:1000, sc-9082, Santa Cruz)
anti-IRF5 (1:1000, ab124792, Abcam)
anti-IRF7 (1:1000; 4920, Cell Signaling)
HRP-conjugated secondary antibody (1:2500; Clean-Blot IP Detection Reagent 21230, Pierce)
anti-β-actin (1:2000; clone ACTBD11B7, sc-81178, Santa Cruz)
HRP-conjugated anti-mouse (1:1000; sc-2314, Santa Cruz)

Immunofluorescense staining:
anti-IRF1 (1:100; ab26109, Abcam)
anti-IRF5 (1:100; ab2932, Abcam)
Alexa Fluor 546-conjugated anti-rabbit (1:400; A10040, Invitrogen)
Alexa Fluor 546-conjugated anti-goat (1:400; A21085, Invitrogen)

ChIP assay:
anti-IRF1 (sc-640X, Santa Cruz)
anti-IRF5 (ab2932, Abcam)
anti-IRF7 (sc-9083X, Santa Cruz)
negative control IgG (sc-2025; Santa Cruz)

Validation

Validation of each primary antibody was based on the information provided by the manufacturer:

CD4 T cell isolation:
Alexa Fluor 488-conjugated anti-CD4 (1:50; clone RPA-T4, 300519, Biolegend): https://www.biolegend.com/nl-nl/products/alexa-fluor-488-anti-human-cd4-antibody-2727
APC-conjugated anti-CD45RA (1:50; clone HI100, 550855, BD Bioscience): https://www.bdbiosciences.com/en-fr/products/reagents/flow-cytometry-reagents/research-reagents/single-color-antibodies-ruo/APC-Mouse-Anti-Human-CD45RA.550855
PE-conjugated anti-CD45RO-PE (200 μg/ml; clone UCHL1, R084301-2, Agilent): https://www.agilent.com/store/en_US/Prod-R084301-2/R084301-2

During culture:
blocking antibodies against dectin-1 (20 μg/ml; clone #259931, MAB1859, R&D Systems): https://www.rndsystems.com/products/human-dectin-1-clec7a-antibody-259931_mab1859
blocking antibodies against IFNα/βR2 (20 μg/ml; clone MMHAR-2, PBL Assay Science): https://www.pblassaysci.com/antibodies/anti-human-ifnar2-antibody-clone-mmhar-2-neutralizing-mab-21385
anti-αvβ1 (10 μg/ml; clone #P5D2, MAB17781, R&D Systems): https://www.rndsystems.com/products/human-integrin-beta1-cd29-antibody-p5d2_mab17781
anti-αvβ3 (10 μg/ml; clone #23C6, MAB3050, R&D Systems): https://www.rndsystems.com/products/human-integrin-alphavbeta3-antibody-23c6_mab3050
anti-αvβ5 (10 μg/ml; clone #P5H9, MAB2528, R&D Systems): https://www.rndsystems.com/products/human-integrin-alphavbeta5-antibody-p5h9_mab2528
anti-αvβ6 (10 μg/ml; clone 10D5, ab77906, Abcam): https://www.abcam.com/integrin-alpha-v--beta-6-antibody-10d5-bsa-and-azide-free-ab77906.html
anti-αvβ8 (10 μg/ml; kind gift from S. L. Nishimura) (not commercially available)

mouse IgG1 isotype control (20 μg/ml; clone MOPC-21, 555746, BD Pharmingen): https://www.bdbiosciences.com/en-au/products/reagents/flow-cytometry-reagents/research-reagents/flow-cytometry-controls-and-lysates/purified-mouse-igg1-isotype-control.555746
mouse IgG2a isotype control (20 μg/ml; clone G155-178, 555571, BD Bioscience): https://www.bdbiosciences.com/en-ca/products/reagents/flow-cytometry-reagents/research-reagents/flow-cytometry-controls-and-lysates/Purified-Mouse-IgG2a,-%CE%BA-Isotype-Control.555571
mouse IgG2b isotype control (20 μg/ml; clone #20116, MAB004, R&D Systems): https://www.rndsystems.com/products/mouse-igg2b-isotype-control_mab004
neutralizing antibodies against IL-1β (5 μg/ml, AF-201-NA, R&D Systems): https://www.rndsystems.com/products/human-il-1beta-il-1f2-antibody_af-201-na
neutralizing antibodies against IL-23 (5 μg/ml, AF1716, R&D Systems): https://www.rndsystems.com/products/human-il-23-p19-antibody_af1716
neutralizing antibodies against IL-12 (5 μg/ml, AF-219-NA, R&D Systems): https://www.rndsystems.com/products/human-il-12-antibody_af-219-na
neutralizing antibodies against IL-27 (5 μg/ml, AF2526, R&D Systems): https://www.rndsystems.com/products/human-il-27-antibody_af2526
neutralizing antibodies TGFβRII (5 μg/ml, AF-241-NA, R&D Systems): https://www.rndsystems.com/products/human-tgf-beta-rii-antibody_af-241-na
normal goat IgG (AB-108-C; R&D Systems) as a control: https://www.rndsystems.com/products/normal-goat-igg-control_ab-108-c

FACS staining:
anti-IRF1 (1:50; ab26109, Abcam): https://www.abcam.com/irf1-antibody-ab26109.html
anti-IRF5 (1:50; ab124792, Abcam): https://www.abcam.com/irf5-antibody-epr6094-ab124792.html
anti-αv (1:50; AF1219, R&D Systems): https://www.rndsystems.com/products/human-mouse-rat-integrin-alphav-cd51-antibody_af1219
anti-β8 (1:50; ab80673, Abcam): https://www.abcam.com/integrin-beta-8-antibody-ab80673.html
anti-BST2 (1:100; NIH AIDS Reagent program #11721)
PE-conjugated anti-rabbit (1:200; 711-116-152, Jackson Immunoresearch): https://www.jacksonimmuno.com/catalog/products/711-116-152/Donkey-Rabbit-IgG-HL-R-Phycoerythrin
Alexa Fluor 488-conjugated anti-goat (1:400; A11055, Invitrogen): https://www.thermofisher.com/antibody/product/Donkey-anti-Goat-IgG-H-L-Cross-Adsorbed-Secondary-Antibody-Polyclonal/A-11055
FITC-conjugated anti-IFNβ (1:20; clone MMHB-3, 21400-3, PBL Assay Science): https://www.pblassaysci.com/antibodies/anti-human-ifn-beta-antibody-clone-mmhb-3-mab-21400 (conjugated no longer available)
FITC-conjugated IgG1κ isotype control mouse Ab (1:250; clone P3.6.2.8.1, 11-4714-81, eBioscience): https://www.thermofisher.com/antibody/product/Mouse-IgG1-kappa-clone-P3-6-2-8-1-Isotype-Control/11-4714-81
APC-conjugated anti-IL-17 (1:25; clone eBio64DEC17, 17-7179-42, eBioscience): https://www.thermofisher.com/antibody/product/IL-17A-Antibody-clone-eBio64DEC17-Monoclonal/17-7179-42
eFluor506-conjugated anti-IL-17 (1:50; clone eBio64DEC17, 69-7179-42, eBioscience): https://www.thermofisher.com/antibody/product/IL-17A-Antibody-clone-eBio64DEC17-Monoclonal/69-7179-42
FITC-conjugated anti-IFN-γ (1:5; clone 25723.11, 25723.11, BD): https://www.bdbiosciences.com/en-us/products/reagents/flow-cytometry-reagents/clinical-discovery-research/single-color-antibodies-ruo-gmp/fitc-mouse-anti-human-ifn.340449
PE-conjugated anti-IL-10 (1:10; clone JES3-9D7, 12-7108-82, Invitrogen): https://www.thermofisher.com/antibody/product/IL-10-Antibody-clone-JES3-9D7-Monoclonal/12-7108-82
Alexa Fluor 647-conjugated anti-RORγt (1:10; clone Q21-559, 563620, BD): https://www.bdbiosciences.com/en-au/products/reagents/flow-cytometry-reagents/research-reagents/single-color-antibodies-ruo/alexa-fluor-647-mouse-anti-human-ror-t.563620
APC-conjugated anti-T-bet (1:10; clone 4B10, 644814, Biolegend): https://www.biolegend.com/en-us/search-results/apc-anti-t-bet-antibody-7120?GroupID=BLG6433
eFluor660-conjugated anti-c-Maf (1:10; clone sym0F1, 50-9855-82, Invitrogen): https://www.thermofisher.com/antibody/product/c-MAF-Antibody-clone-sym0F1-Monoclonal/50-9855-82
APC-conjugated anti-IL-1R1 (1:10; FAB269A, R&D Systems): https://www.rndsystems.com/products/human-il-1-ri-apc-conjugated-antibody_fab269a
APC-conjugated anti-GM-CSF (1:10; clone BVD2-21C11, 502310, Biolegend): https://www.biolegend.com/en-gb/products/apc-anti-human-gm-csf-antibody-7777
Alexa Fluor 647-conjugated mouse IgG2b (1:5; clone 27-35, 558713, BD): https://www.bdbiosciences.com/en-au/products/reagents/flow-cytometry-reagents/research-reagents/single-color-antibodies-ruo/alexa-fluor-647-mouse-igg2b-isotype-control.558713
APC-conjugated mouse IgG1 (10 μg/ml; clone P3.6.2.8.1, 17-4714-42, Invitrogen): https://www.thermofisher.com/antibody/product/Mouse-IgG1-kappa-clone-P3-6-2-8-1-Isotype-Control/17-4714-42
eFluor660-conjugated mouse IgG2b (10 μg/ml; clone eBMG2b, 50-4732-82, Invitrogen): https://www.thermofisher.com/antibody/product/Mouse-IgG2b-kappa-clone-eBMG2b-Isotype-Control/50-4732-82
APC-conjugated goat IgG (1:5; IC108A, R&D Systems): https://www.rndsystems.com/products/goat-igg-apc-conjugated-antibody_ic108a
APC-conjugated rat IgG2a (2.5 μg/ml; 402305, Biolegend): https://www.biolegend.com/fr-fr/products/apc-rat-igg2a-lambda-isotype-control-antibody-19307

ELISA:
anti-pan TGFβ (2 μg/ml, coating Ab; clone 1D11, MAB1835, R&D Systems): https://www.rndsystems.com/products/tgf-beta1-2-3-antibody-1d11_mab1835
biotinylated anti-TGFβ1 (0.2 μg/ml, detecting Ab; BAF240, R&D Systems): https://www.rndsystems.com/products/tgf-beta1-biotinylated-antibody_baf240

Immunoblotting:
anti-IRF1 (1:1000; 8478, Cell Signaling): https://www.cellsignal.com/products/primary-antibodies/irf-1-d5e4-xp-rabbit-mab/8478
anti-IRF3 (1:1000, sc-9082, Santa Cruz): https://www.scbt.com/p/irf-3-antibody-fl-425
anti-IRF5 (1:1000, ab124792, Abcam): https://www.abcam.com/irf5-antibody-epr6094-ab124792.html
anti-IRF7 (1:1000; 4920, Cell Signaling): https://www.cellsignal.com/products/primary-antibodies/irf-7-antibody/4920
HRP-conjugated secondary antibody (1:2500; Clean-Blot IP Detection Reagent 21230, Pierce): https://www.thermofisher.com/order/

catalog/product/21230
anti-β-actin (1:2000; clone ACTBD11B7, sc-81178, Santa Cruz): https://www.scbt.com/p/beta-actin-antibody-actbd11b7?requestFrom=search
HRP-conjugated anti-mouse (1:1000; sc-2314, Santa Cruz): https://www.scbt.com/p/donkey-anti-mouse-igg-hrp

Immunofluorescense staining:
anti-IRF1 (1:100; ab26109, Abcam): https://www.abcam.com/irf1-antibody-ab26109.html
anti-IRF5 (ab2932, Abcam): https://www.abcam.com/irf5-antibody-ab2932.html
Alexa Fluor 546-conjugated anti-rabbit (1:400; A10040, Invitrogen): https://www.thermofisher.com/antibody/product/Donkey-anti-Rabbit-IgG-H-L-Highly-Cross-Adsorbed-Secondary-Antibody-Polyclonal/A10040
Alexa Fluor 546-conjugated anti-goat (1:400; A21085, Invitrogen): https://www.thermofisher.com/antibody/product/Rabbit-anti-Goat-IgG-H-L-Cross-Adsorbed-Secondary-Antibody-Polyclonal/A-21085

ChIP assay:
anti-IRF1 (sc-640X, Santa Cruz): https://www.scbt.com/p/irf-1-antibody-m-20
anti-IRF5 (ab2932, Abcam): https://www.abcam.com/irf5-antibody-ab2932.html
anti-IRF7 (sc-9083X, Santa Cruz): https://www.scbt.com/p/irf-7-antibody-h-246
negative control IgG (sc-2025; Santa Cruz): https://www.scbt.com/p/normal-mouse-igg

Eukaryotic cell lines

Policy information about cell lines

Cell line source(s)	HEK-Blue™ TGF-β reporter cells: Invivogen
Authentication	This cell line was not authenticated
Mycoplasma contamination	The cell line was tested negative for mycoplasma contamination.
Commonly misidentified lines (See ICLAC register)	No misidentified lines are used.

Human research participants

Policy information about studies involving human research participants

Population characteristics	Healthy donors: F 27 yrs, F 47 yrs, M 27 yrs, M 61 yrs; acute, untreated CD donors: F 20 yrs, F 56 yrs, M 40 yrs, M 50 yrs; treated CD donors: F 37 yrs, F 59 yrs, M 27 yrs, M 70 yrs.
Recruitment	Patients that were seen at the IBD clinic of Amsterdam UMC, location AMC, and were diagnosed with Crohn's disease, but had not yet started treatment were selected for the 'acute, untreated' group, while patients that had been diagnosed previously and had gone into remission as a result of treatment were selected for the 'treated' group. We had no criteria on age or gender. We tried to match the age and gender of healthy subjects to the CD donors.
Ethics oversight	This study was done in accordance with the ethical guidelines of the Amsterdam UMC, location AMC and human material was obtained in accordance with the AMC Medical Ethics Review Committee (i.e. Institutional Review Committee) according to the Medical Research Involving Human Subjects Act. Buffy coats obtained after blood donation (Sanquin) are not subjected to informed consent according to the Medical Research Involving Human Subjects Act and the AMC Medical Ethics Review Committee. Blood obtained from healthy volunteers was covered by the BACON protocol. Patients with active Crohn's disease were recruited at the IBD clinic of Amsterdam UMC, location AMC. After providing written informed consent, an additional blood sample was drawn in addition to routine blood draws. The project was covered by the Future-IBD biobank protocol. All samples were handled anonymously.

Note that full information on the approval of the study protocol must also be provided in the manuscript.

Flow Cytometry

Plots

Confirm that:

☒ The axis labels state the marker and fluorochrome used (e.g. CD4-FITC).

☒ The axis scales are clearly visible. Include numbers along axes only for bottom left plot of group (a 'group' is an analysis of identical markers).

☒ All plots are contour plots with outliers or pseudocolor plots.

☒ A numerical value for number of cells or percentage (with statistics) is provided.

Methodology

Sample preparation	FACS analyses:<br>Human monocyte-derived dendritic cells were differentiated from monocytes isolated from buffy coats.

CD4+ T cells were isolated by negative selection from buffy coats, after which naive and memory T cells were separated by positive selection for CD45RO, and used in cocultures with moDCs. Cells were fixed with para-formaldehyde and permeabilized using either saponin or MeOH.

FACS sorting:
moDCs were transfected with a pCG-BST2-IRES-eGFP expression plasmid and sorted based on low and high eGFP.
CD4+ T cells were isolated by negative selection from blood donated by healthy donors or Crohn's disease patients, stimulated for 6 h with PMA/ionomycin, fixed with para-formaldehyde, stained with APC- or eFluor506-conjugated anti-IL-17 and sorted based on IL-17- and IL-17+.

Instrument	FACS Canto (BD Biosciences), FACS Calibur (BD Biosciences) and FACSAria IIu Cell Sorter (BD Biosciences)
Software	FlowJo 10.7.1
Cell population abundance	The complete post-sort fraction was relevant and used for further experiments.
Gating strategy	FACS analyses:<br>moDCs and T cells were gated on live cells and size based on FCS-A vs. SSC-A. Differentiated T cells were further gated on single cells based on FSC-H vs. FSC-A.<br><br>FACS sorting:<br>For sorting, moDCs cells were further gated on single cells based on FSC-H vs. FSC-W and SSC-H vs. FCS-W. These single cells were plotted GFP vs. a dump channel and the populations were sorted in GFP- and GFP++.<br>For sorting, T cells cells were further gated on single cells based on FSC-H vs. FSC-W and SSC-H vs. FCS-W. These single cells were plotted IL-17 APC vs. a dump channel or IFNg FITC and the populations were sorted for IL-17+, IL-17+IFNg- or IL-17+IFNg +.

☒ Tick this box to confirm that a figure exemplifying the gating strategy is provided in the Supplementary Information.
