# Supplementary material for: Fungal sensing by dectin-1 directs the non-pathogenic polarization of TH17 cells through balanced type I IFN responses in human DCs
Source: Nat Immunol. 2022 Dec 1;23(12):1735–48. doi: 10.1038/s41590-022-01348-2 (PMC9747615; doi:10.1038/s41590-022-01348-2)
Supplement: Source Data Fig. 3 — Unprocessed immunoblots. [file 41590_2022_1348_MOESM7_ESM.pdf]

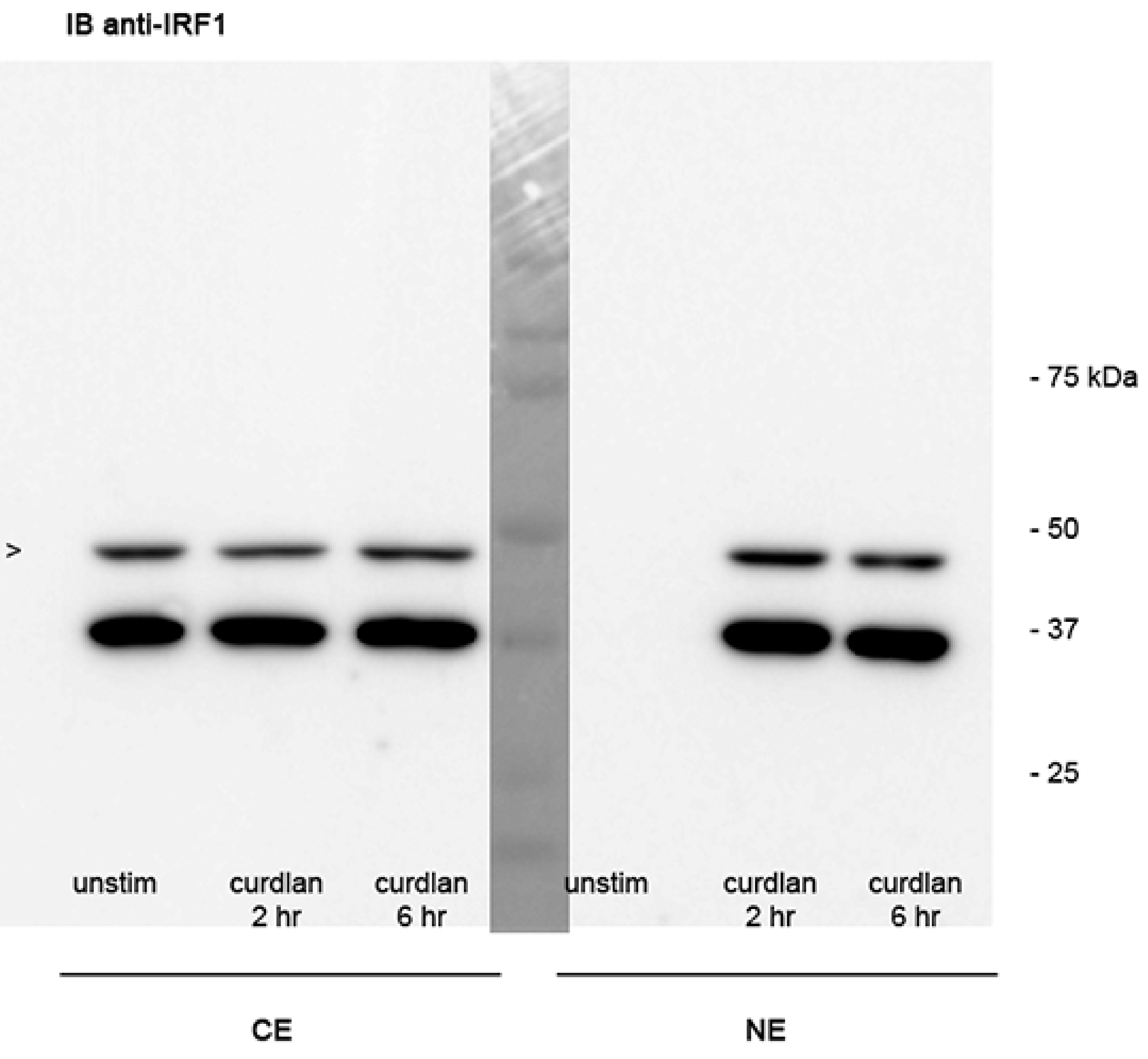
IB anti-IRF1
>
- 75 kDa
- 50
- 37
- 25
unstim
curdlan 2 hr
curdlan 6 hr
unstim
curdlan 2 hr
curdlan 6 hr
CE
NE

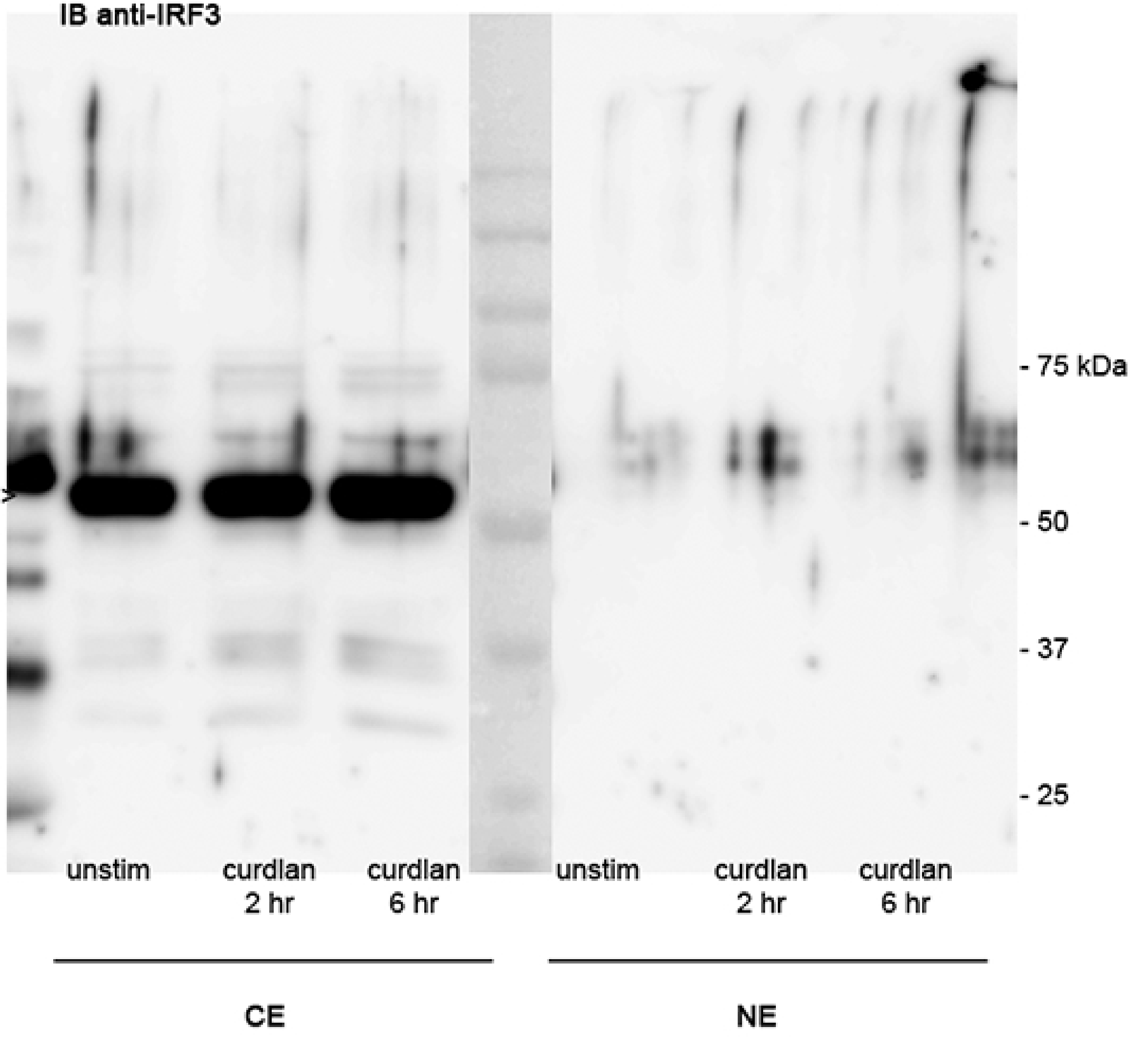
IB anti-IRF3
- 75 kDa
- 50
- 37
- 25
unstim
curdlan 2 hr
curdlan 6 hr
unstim
curdlan 2 hr
curdlan 6 hr
CE
NE

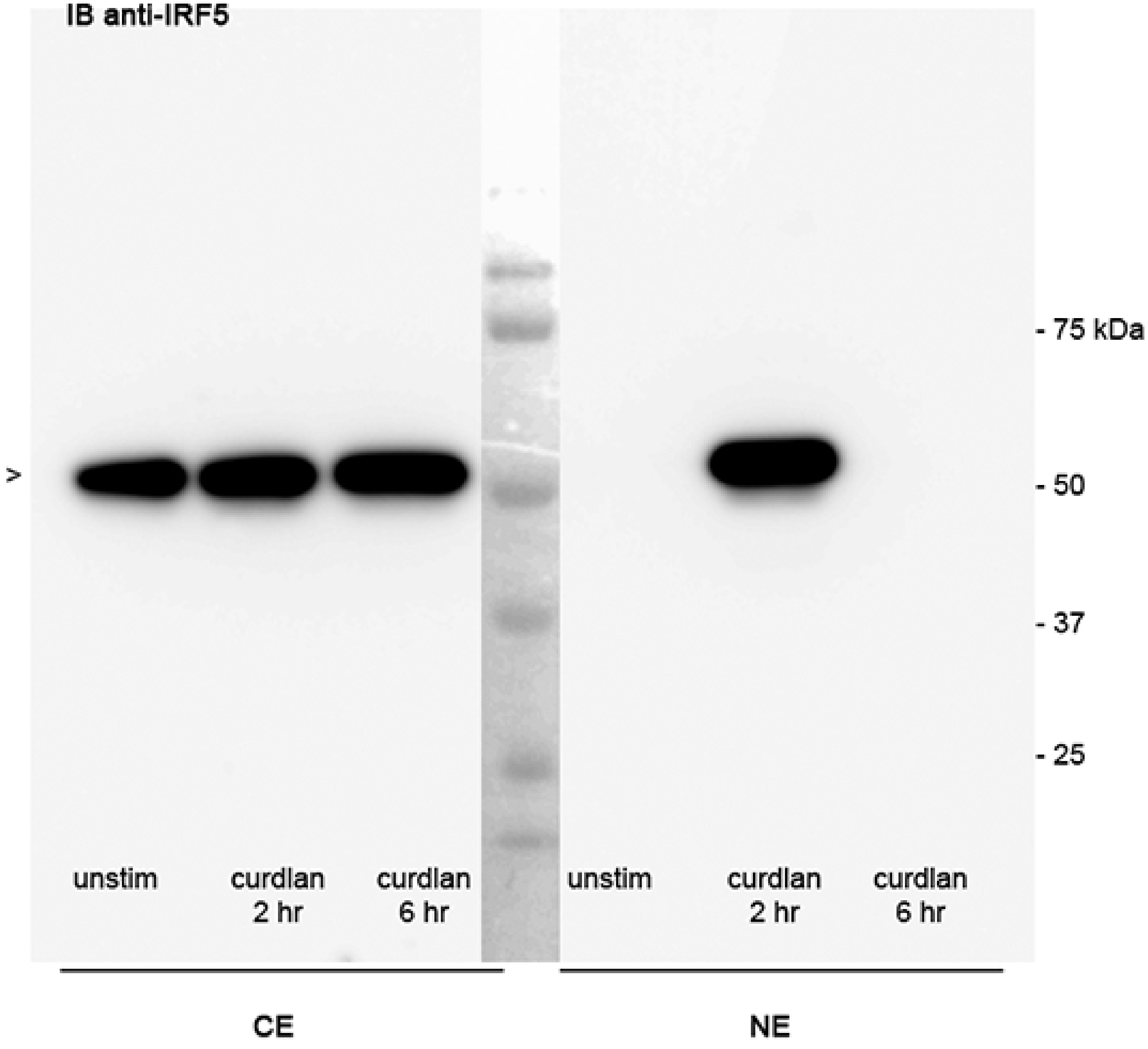
IB anti-IRF5
>
- 75 kDa
- 50
- 37
- 25
unstim
curdlan 2 hr
curdlan 6 hr
unstim
curdlan 2 hr
curdlan 6 hr
CE
NE

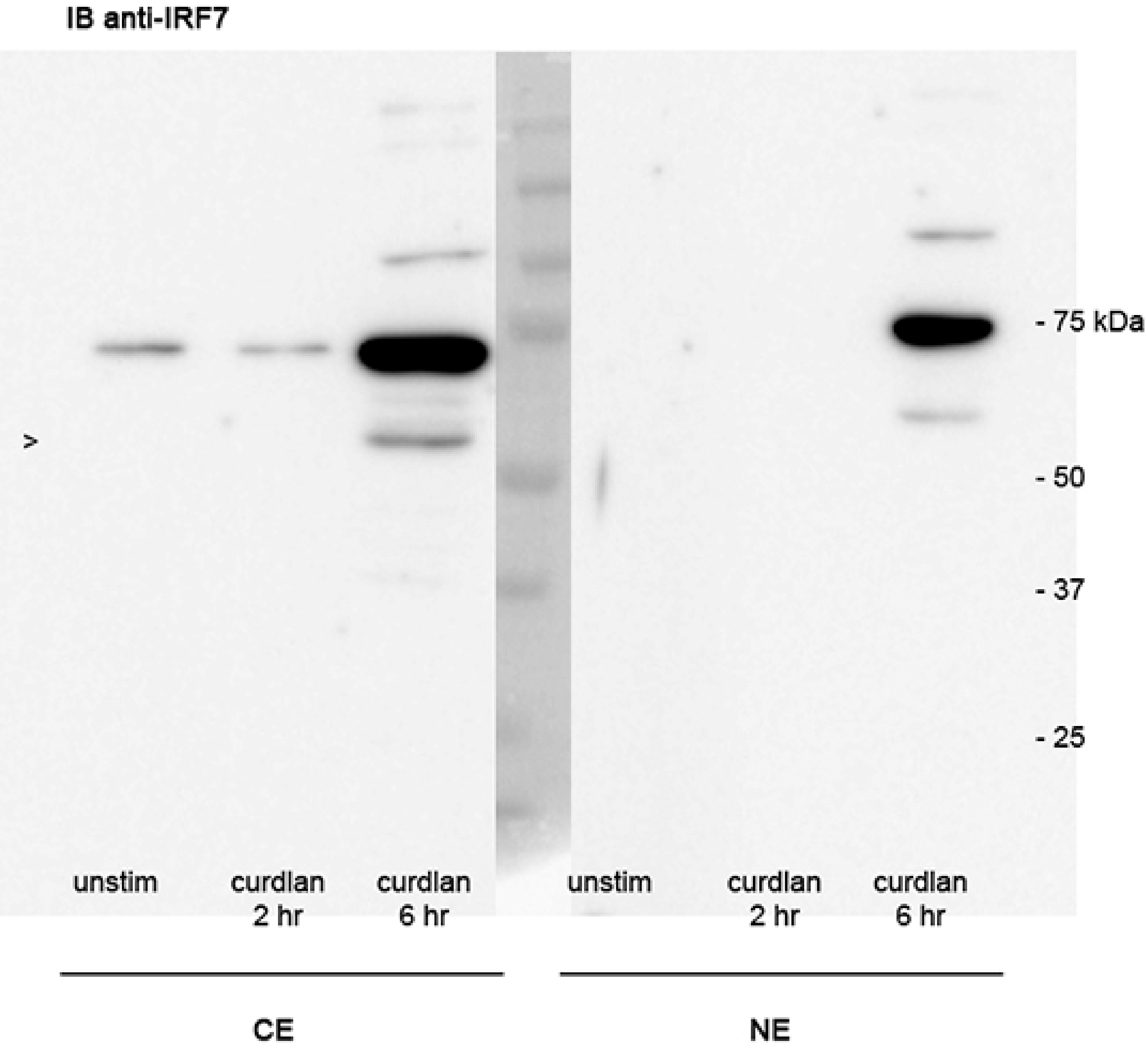
IB anti-IRF7
>
- 75 kDa
- 50
- 37
- 25
unstim
curdlan 2 hr
curdlan 6 hr
unstim
curdlan 2 hr
curdlan 6 hr
CE
NE

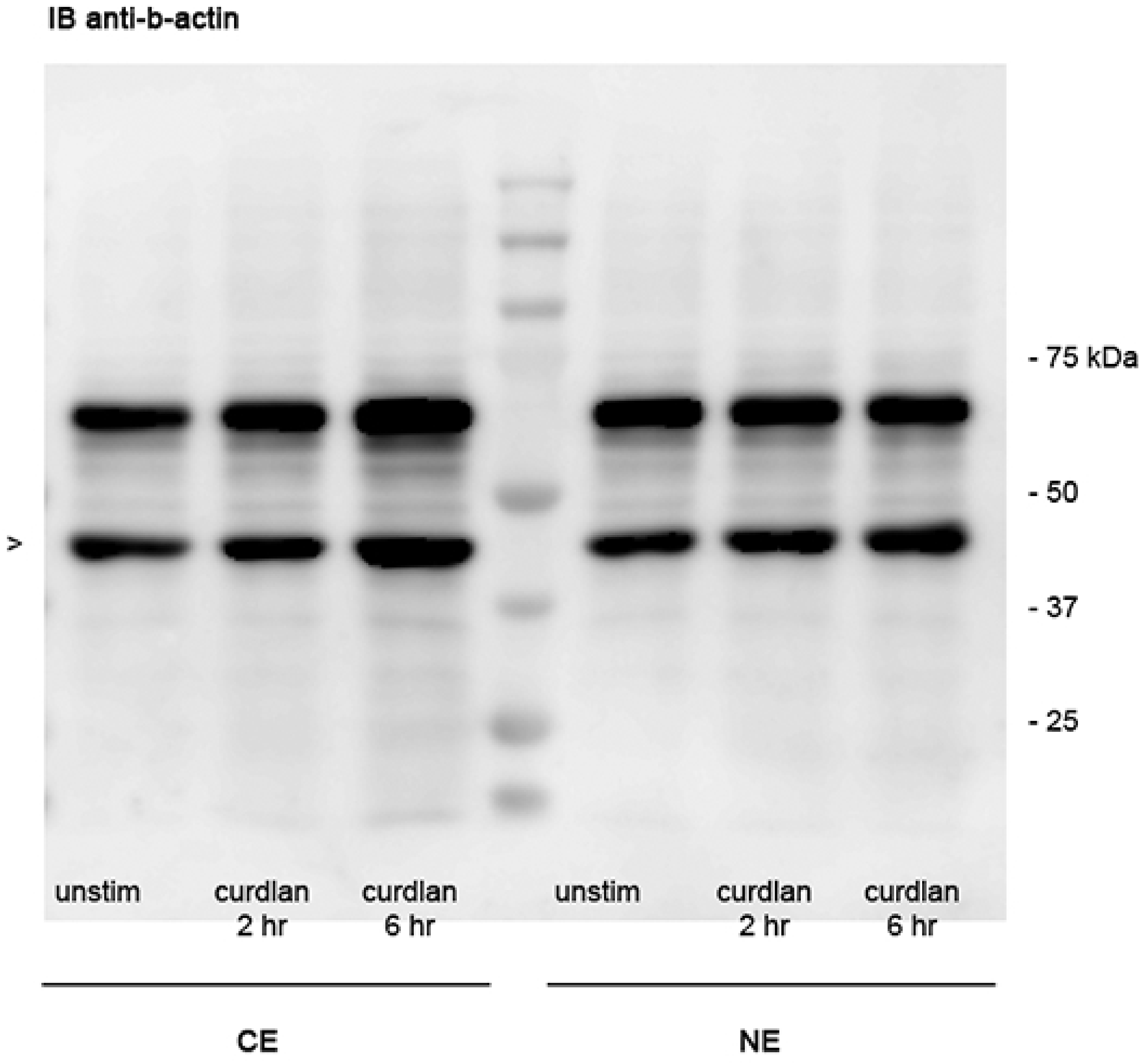
IB anti-b-actin
- 75 kDa
- 50
- 37
- 25
>
unstim
curdlan 2 hr
curdlan 6 hr
unstim
curdlan 2 hr
curdlan 6 hr
CE
NE
